# An Item Response Theory–Informed Strategy to Model Total Score Data from Composite Scales

**DOI:** 10.1208/s12248-021-00555-3

**Published:** 2021-03-16

**Authors:** Gustaf J. Wellhagen, Sebastian Ueckert, Maria C. Kjellsson, Mats O. Karlsson

**Affiliations:** Pharmacometrics Research Group, Department of Pharmacy, Uppsala University, Box 580, 751 23 Uppsala, Sweden

**Keywords:** bounded integer model, composite scale data, IRT-informed total score analysis, total score analysis

## Abstract

Composite scale data is widely used in many therapeutic areas and consists of several categorical questions/items that are usually summarized into a total score (TS). Such data is discrete and bounded by nature. The gold standard to analyse composite scale data is item response theory (IRT) models. However, IRT models require item-level data while sometimes only TS is available. This work investigates models for TS. When an IRT model exists, it can be used to derive the information as well as expected mean and variability of TS at any point, which can inform TS-analyses. We propose a new method: IRT-informed functions of expected values and standard deviation in TS-analyses. The most common models for TS-analyses are continuous variable (CV) models, while bounded integer (BI) models offer an alternative that respects scale boundaries and the nature of TS data. We investigate the method in CV and BI models on both simulated and real data. Both CV and BI models were improved in fit by IRT-informed disease progression, which allows modellers to precisely and accurately find the corresponding latent variable parameters, and IRT-informed SD, which allows deviations from homoscedasticity. The methodology provides a formal way to link IRT models and TS models, and to compare the relative information of different model types. Also, joint analyses of item-level data and TS data are made possible. Thus, IRT-informed functions can facilitate total score analysis and allow a quantitative analysis of relative merits of different analysis methods.

**Supplementary Information:**

The online version contains supplementary material available at 10.1208/s12248-021-00555-3.

## BACKGROUND

Composite scales are commonly used in many disease areas, such as CNS disorders and autoimmune diseases (1). Often these scales were developed for diagnosis, but in lack of reliable biomarkers they also function as clinical endpoints to evaluate disease progression and treatment efficacy. Such scales consist of several questions/items that are summarized to a total score (TS), often the sum of the item scores. The resulting TS is discrete and bounded.

Item-level data contains all the information collected, and therefore adequately designed item response theory (IRT) models are the most informative way to analyse composite scale data (2). These models include item characteristic curves (ICCs) for each item and handle correlation between items through one or several latent variables. However, IRT models may be complex to develop, require large datasets, include many parameters, and take long time to estimate. Most importantly, they cannot be used if data on the item level is not available, which is the case we investigate here.

When the item-level data is not available, the TS can be modelled—which is the focus of this work. For this, several approaches are used such as continuous variable (CV), bounded integer (BI) (3), beta regression (4), or coarsened grid models (5)—here we investigate CV and BI models. None of these methods uses the information from item-level data. However, if there exists an IRT model for the same composite scale as the TS data, it might be used to inform TS-analyses. Bounded data has lower variability at the boundaries, as these impose natural limits to the outcome. Thus, a homoscedastic error, as is typical in CV models, is not the best description of the variability. Instead, the mean and variability at each latent variable value in an IRT model can be computed through the ICCs. Therefore, an IRT model can yield the expected variability at any predicted TS value.

An example of a composite scale is the Movement Disorder Society–sponsored revision of the Unified Parkinson’s Disease Rating Scale (MDS-UPDRS) (6). There are four parts to MDS-UPDRS: nonmotor and motor aspects of experiences of daily living, motor examination, and motor complications, totally 68 items. Parkinson’s disease is heterogeneous and different aspects of the disease progress at different rates (7, 8). Since drug effects are unlikely to affect all items equally, drug development within Parkinson’s disease is often focused on a part of the whole MDS-UPDRS scale to gain power; items are typically reassigned as nonmotor-, motor-, or tremor-related, where the total subscore is used as an endpoint.

Indeed, recent work has shown that the disease progressions of nonmotor, motor, and tremor complications can be modelled separately in an IRT framework (9). In this work, we focus only on the motor subscore which is typically the most sensitive to drug effects since it has the fastest progression rate (10)—it is also the largest subscale of MDS-UPDRS.

IRT-informed TS-analysis, presented in this work, is a new method to improve fit and parameter precision as well as description of variability and disease progression in TS-analyses. The idea is that modellers dealing with TS data from a composite scale could gain information from existing IRT models for that composite scale. To this end, IRT-informed link functions between one previously published IRT model and different TS models, and link functions describing the standard deviation (SD) are evaluated on both simulated and real data within Parkinson’s disease.

## METHODS

### Models

Eight different models for total score data were evaluated in this work: four CV models and four BI models (standard models, fully IRT-informed models for both mean and SD, and partially IRT-informed models for either mean or SD). They operate on the TS scale, Z scale (latent variable of BI), and/or the Ψ scale (latent variable of IRT). The TS is bounded, while Ψ, Z ∈ *ℝ*. A descriptive summary of the models is shown in Table I.

**Table I:** Properties of the Investigated Models

Model	Description	Scale	Standard deviation
S-CV	Standard CV model	TS	Homoscedastic (estimated θ)
SDI-CV	Partially (SD) IRT-informed CV model		Heteroscedastic (fixed *SD*(*Y*| Ψ))
MI-CV	Partially (mean) IRT-informed CV model	Ψ	Homoscedastic (estimated θ)
I-CV	Fully IRT-informed CV model		Heteroscedastic (fixed *SD*(*Y*| Ψ))
S-BI	Standard BI model	Z	Homoscedastic (estimated θ)
SDI-BI	Partially (SD) IRT-informed BI model		Heteroscedastic (fixed *SD*(*Y*| Ψ))
MI-BI	Partially (mean) IRT-informed BI model	Ψ	Homoscedastic (estimated θ)
I-BI	Fully IRT-informed BI model		Heteroscedastic (fixed *SD*(*Y*| Ψ))

*BI* bounded integer, *CV* continuous variable, *IRT* item response theory, *Ψ* latent variable of IRT, *SD*(*Y*| Ψ) standard deviation from IRT model, *TS* total score, *Z* latent variable of BI

#### Continuous Variable Models

Under the standard CV model with homoscedastic error (S-CV), the observation *j* for subject *i* at time *t*_*ij*_ follows:

$$ {\displaystyle \begin{array}{c}{Y}_{ij}=f\left(\varTheta, {\eta}_i,{t}_{ij},{X}_i\right)+{\varepsilon}_{ij}\\ {}{\eta}_i\sim N\left(0,{\omega}^2\right)\\ {}{\varepsilon}_{ij}\sim N\left(0,{\sigma}^2\right)\end{array}} $$

where *Θ* are fixed effect parameters, *η*_*i*_ the random effects of the inter-individual (IIV), *X*_*i*_ the covariates, *ε*_*ij*_ the residual unexplained variability (RUV), *ω*^2^ the variance of the IIV, and σ^2^ the variance of the RUV.

The fully IRT-informed CV model (I-CV) is expressed as:

$$ {\displaystyle \begin{array}{c}{\Psi}_{ij}=h\left(\varTheta, {\eta}_i,{t}_{ij},{X}_i\right)\\ {}{Y}_{ij}={pn}_1\left({\Psi}_{ij}\right)+{\varepsilon}_{ij}\cdotp {pn}_2\left({\Psi}_{ij}\right)\\ {}\begin{array}{c}{\eta}_i\sim N\left(0,{\omega}^2\right)\\ {}{\varepsilon}_{ij}\sim N\left(0,1\right)\end{array}\end{array}} $$

where Ψ_*ij*_ is a latent variable described by the function *h*(·), which is typically a function for disease progression and treatment effects on the latent variable scale, and *pn*_*1*_ as well as *pn*_*2*_ are predetermined polynomials with coefficients calibrated to theoretical expectations (see “IRT-informed functions” below). All other variables maintain their definition from above.

Furthermore, we define the partially IRT-informed CV model for the mean (MI-CV) as

$$ {Y}_{ij}={\mathrm{p}n}_1\left({\Psi}_{ij}\right)+{\varepsilon}_{ij} $$

and for the SD (SDI-CV) as

$$ {Y}_{ij}=f\left(\varTheta, {\eta}_i,{t}_{ij},{X}_i\right)+{\varepsilon}_{ij}\cdotp {pn}_2\left({\Psi}_{ij}\right). $$

#### Bounded Integer Models

The standard BI model (S-BI) is a discrete data model for bounded outcomes, where the probability of an individual *i* to have the score *k* at time *t*_*ij*_ is:

$$ P\left({Y}_{ij}=k\right)=\phi \left(\frac{Z_{\frac{k}{n}}-f\left(\uptheta, {\upeta}_i,{t}_{ij},{X}_i\right)\ }{g\left(\upsigma, {\upeta}_i,{t}_{ij},{X}_i\right)\ }\right)-\phi \left(\frac{Z_{\frac{k-1}{n}}-f\left(\uptheta, {\upeta}_i,{t}_{ij},{X}_i\right)\ }{g\left(\upsigma, {\upeta}_i,{t}_{ij},{X}_i\right)\ }\right)\kern0.5em {\eta}_i\sim N\left(0,{\omega}^2\right) $$

where ϕ is the cumulative distribution function for the standard normal distribution, Z_k/n_ and Z_(k-1)/n_ are the cut points between categories *k* and *k-1* defined through the probit function for an *n*-category scale, *f*(·) is the function for the mean, and *g*(·) the function for the variance on the probit scale. The probability mass function across all scores adds to 1. For all BI models, the special cases for the first and last categories *(k = 1*, *k = n)* apply:

$$ {\displaystyle \begin{array}{c}P\left({Y}_{ij}=1\right)=\phi \left(\frac{Z_{\frac{1}{n}}-f\left(\uptheta, {\upeta}_i,{t}_{ij},{X}_i\right)\ }{g\left(\upsigma, {\upeta}_i,{t}_{ij},{X}_i\right)\ }\right)\\ {}P\left({Y}_{ij}=n\right)=1-\phi \left(\frac{Z_{\frac{n-1}{n}}-f\left(\uptheta, {\upeta}_i,{t}_{ij},{X}_i\right)\ }{g\left(\upsigma, {\upeta}_i,{t}_{ij},{X}_i\right)\ }\right)\end{array}} $$

The fully IRT-informed BI model (I-BI) is expressed as:

$$ {\displaystyle \begin{array}{c}{\Psi}_{ij}=h\left(\varTheta, {\eta}_i,{t}_{ij},{X}_i\right)\\ {}P\left({Y}_{ij}=k\right)=\phi \left(\frac{Z_{\frac{k}{n}}-{pn}_3\left({\Psi}_{ij}\right)\ }{pn_4\left({\varPsi}_{ij}\right)\ }\right)-\phi \left(\frac{Z_{\frac{k-1}{n}}-{pn}_3\left({\Psi}_{ij}\right)}{pn_4\left({\varPsi}_{ij}\right)\ }\right)\\ {}{\eta}_i\sim N\left(0,{\omega}^2\right)\end{array}} $$

where *pn*_*3*_ and *pn*_*4*_ are distinct polynomials from *pn*_*1*_ and *pn*_*2*_. Similar to the IRT-informed CV models, we also define the partial versions for the mean (MI-BI):

$$ P\left({Y}_{ij}=k\right)=\phi \left(\frac{Z_{\frac{k}{n}}-{pn}_3\left({\Psi}_{ij}\right)\ }{g\left(\upsigma, {\upeta}_i,{t}_{ij},{X}_i\right)\ }\right)-\phi \left(\frac{Z_{\frac{k-1}{n}}-{pn}_3\left({\Psi}_{ij}\right)\ }{g\left(\upsigma, {\upeta}_i,{t}_{ij},{X}_i\right)\ }\right) $$

and the SD (SDI-BI):

$$ P\left({Y}_{ij}=k\right)=\phi \left(\frac{Z_{\frac{k}{n}}-f\left(\uptheta, {\upeta}_i,{t}_{ij},{X}_i\right)\ }{pn_4\left({\varPsi}_{ij}\right)\ }\right)-\phi \left(\frac{Z_{\frac{k-1}{n}}-f\left(\uptheta, {\upeta}_i,{t}_{ij},{X}_i\right)\ }{pn_4\left({\varPsi}_{ij}\right)\ }\right) $$

### Derivation of IRT-Informed Functions

Based only on the ICCs (no new data is required) from a published IRT model (9) for the MDS-UPDRS motor subscale, analytic links allowing a direct translation between the latent variable of the IRT model and the expected mean and SD were derived on the TS scale for the CV model and on the Z score scale for the BI model. For the CV model, the expected value and the variance of the total score as a function of the latent variable Ψ were calculated according to:

$$ {\displaystyle \begin{array}{c}E\left(Y|\Psi \right)=\sum \limits_{m=1}^M\sum \limits_{s=1}^{S_m}s\cdotp P\left({Y}_m=s|\Psi \right)\\ {} Var\left(Y|\Psi \right)=\sum \limits_{m=1}^M\sum \limits_{s=1}^{S_j}{\left(s-E\left(Y|\Psi \right)\right)}^2\cdotp P\left({Y}_m=s|\Psi \right)\end{array}} $$

where *P*(*Y*_*m*_ = *s* │ Ψ) is the response probability for item *m* and category *s* given a particular latent variable value (from the published ICCs), *M* is the number of items in the MDS-UPDRS motor subscale, and *S*_*m*_ is the number of categories for item *m*. The analytic link for the BI model is given in Supplemental Material 1.

In a second step, the analytically derived link functions for the expected value and the standard deviation ($SD\left(Y|\Psi \right)=\sqrt{Var\left(Y|\Psi \right)}$) were approximated using empirical Chebyshev polynomials across the whole disease range for the motor subscale, i.e. Ψ ranged from − 4 to + 8. Starting from 1, the polynomial degree was increased until the maximum deviation between analytic link and polynomial approximation was below a pre-specified tolerance (0.01 and 0.01 for the expected value and SD of the CV model, respectively, and 0.01 and 0.005 for the corresponding BI values).

All calculations were implemented in R and were made available in the package piraid (11) (for full details on how to automatically create polynomials and control streams for a given composite scale, see Supplemental Material 2, where relevant piraid R code is supplied with some illustrative examples).

For the fully IRT-informed models (I-CV, I-BI), link functions were used between Ψ and mean TS/Z score as well as between Ψ and SD. The partially IRT-informed models illustrate how much improvement either the description of mean or SD brings, respectively, but if the IRT-informed approach is taken, it should of course be natural to use the fully IRT-informed models. For the partially (mean) IRT-informed models (MI-CV, MI-BI), only the link between Ψ and mean TS/Z score was used. For the partially (SD) IRT-informed models (SDI-CV, SDI-BI), link functions were only implemented between mean TS/Z score and *SD*(*Y*| Ψ). All these polynomials as well as examples of *h*(·) functions are given in Supplemental Material 3, and NONMEM control stream examples for I-CV and I-BI are given in Supplemental Material 4. The NONMEM control stream for the IRT simulation model, including the ICC parameters, is shown in Supplemental Material 5.

#### Information Content

Fisher information, often used in optimal design to understand the compare different study designs, can also be used to appreciate what parts of the data are considered most informative under a given model. For that purpose, the Fisher information for Ψ was calculated for the IRT, the I-CV, and the I-BI model according to the equation presented in Supplemental Material 6. The resulting information, as a function of Ψ, was visualized graphically and the information content from a S-CV model with homoscedastic residual error model was overlaid. The calculation of information content is conditioned of the model being an adequate description of the data, which is the case for the IRT, I-CV, and I-BI model, but not the S-CV model.

### Applications

#### Applications to Simulated Data

The approach of IRT-informed TS-analysis was investigated on two simulated datasets. First, one simulation of 1000 individuals at 10 occasions was performed with IRT latent variable baseline at 0.654 and no disease progression, meaning that subjects had the same value of the latent variable throughout the study. This dataset was analysed with S-CV and S-BI models with homoscedastic SD—both with and without an associated log-normally distributed IIV. Alternatively, *SD*(*Y*| Ψ) was used to describe the SD, meaning no parameter relating to SD was estimated. Additional complexity on top of *SD*(*Y*| Ψ) was also evaluated, which allowed for higher or lower (non-negative) SD but retaining the shape of *SD*(*Y*| Ψ): multiplication by a parameter (bound to be positive), lognormal IIV in SD, multiplication by a parameter with lognormal IIV, and lastly addition of a parameter (bound to be positive) with lognormal IIV.

Second, another simulation of 1000 individuals at 10 occasions was performed with IRT latent variable baseline at 0.654 and a linear disease progression of 0.449 year^−1^. The dataset was again analysed with CV and BI models both with homoscedastic SD and *SD*(*Y*| Ψ), this time in combination with the disease progression model. Both linear disease progression on TS or Z and linear disease progression on Ψ through *E*(*Y*| Ψ) were evaluated.

#### Parameter Precision and Bias

Models that use the IRT-informed link functions allow estimation of baseline and disease progression parameters on the same scale as the IRT model. It is therefore of interest to assess whether such parameters can be accurately estimated. For each studied scenario, containing studies of 1000 subjects over 10 occasions, 100 datasets were simulated and parameter estimated based on the simulated data. In each of these, the IRT model was used as simulation model and the I-CV, MI-CV, I-BI, and MI-BI models were used for parameter estimation. The precision was evaluated through the width of the percentiles of the estimated parameter values and the bias through the percent difference of the mean parameter estimate from the true parameter value.

#### Application to Real Data

The real dataset was the same as in the previously described (3), and came from the Parkinson’s Progression Markers Initiative (PPMI) (www.ppmi-info.org) (12) with 428 de novo patients with Parkinson’s disease who were followed up to 48 months, totaling 2720 observations. Both CV and BI models were used to fit the data, where inclusion of *E*(*Y*| Ψ) and/or *SD*(*Y*| Ψ) was evaluated, and the base model structure (in both standard and IRT-informed models) was the same as previously reported (3, 9), with disease progression described through a linear slope and the effect of medication included as an offset effect:

$$ h\left(\cdotp \right)={\theta}_1+{\theta}_2\cdotp t-{\theta}_3\cdotp {X}_1 $$

where *θ*_1_ is the baseline, *θ*_2_ the slope, *θ*_3_ the effect of medication, *X*_1_ the covariate of medication (1/0), and *t* time. The same additional complexity models for *SD*(*Y*| Ψ) as described for the simulated datasets were evaluated, but the base model structure was always the same. Improved model fit was assessed by objective function value (OFV) or Akaike information criterion (AIC), and visual predictive checks (VPC).

### Software

Nonlinear mixed-effects modelling was performed with NONMEM version 7.4 (ICON Development Solutions, Ellicott City, MD), executed through PsN version 4.9 (13, 14). The Laplacian estimation method with η-ε interaction was used for all the CV models, while BI models were estimated with stochastic approximation expectation maximization (SAEM). To be able to compare the OFV between different models, importance sampling with expectation only was added in a second estimation step. Graphics were made with R version 3.6.2 (15) and the polynomials were calculated in piraid (11).

## RESULTS

### Derivation of IRT-Informed Functions

**Fig. 1:**
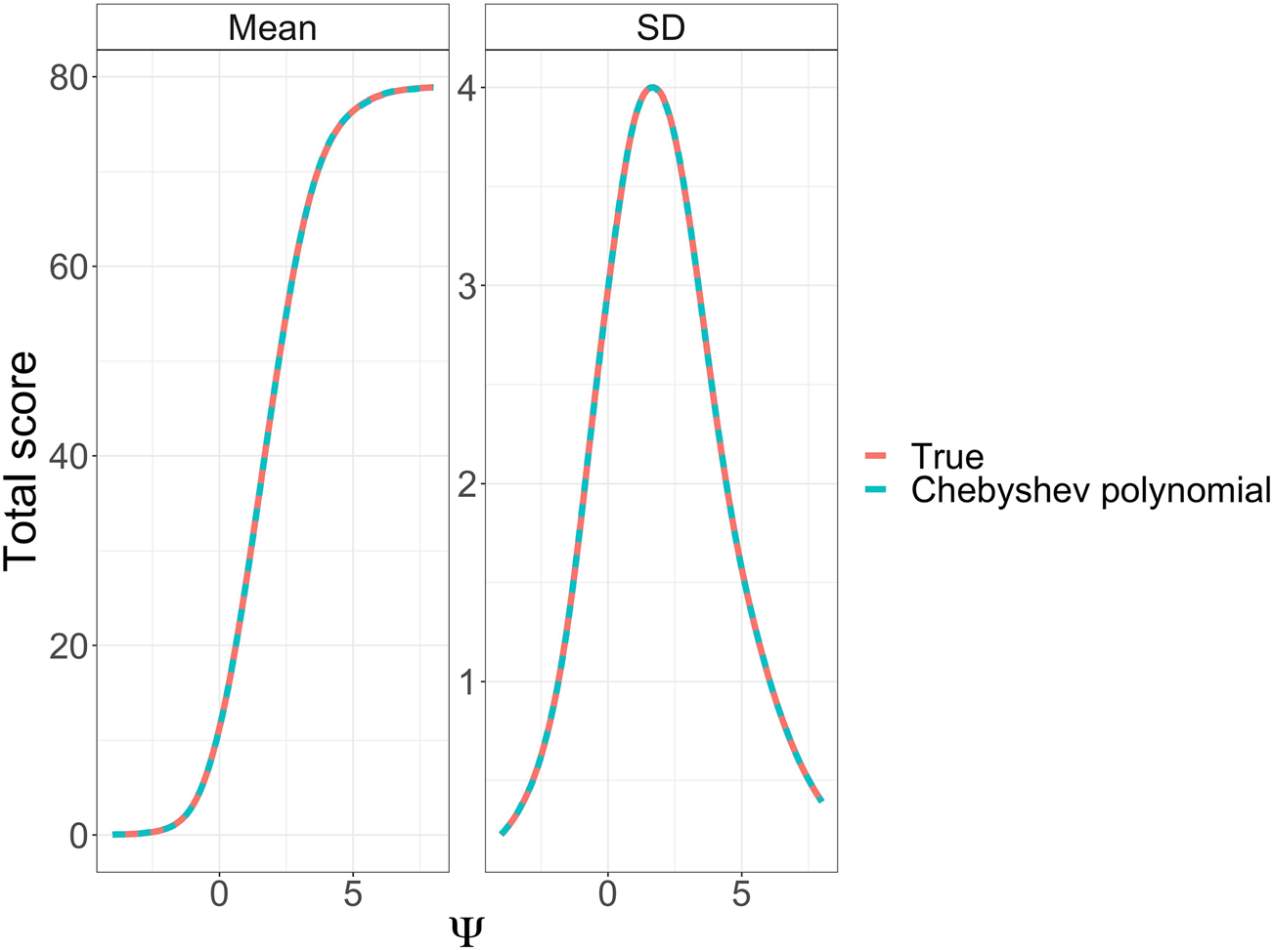
Mean TS and SD as a function of the latent variable (Ψ) for a CV model

High-order polynomials (12–23) were sufficient to adequately map Ψ to TS and Z scales. As Ψ increased from low to high values, the TS increased with an S-shape, as seen in Fig. 1. The SD of TS showed strong deviation from homoscedasticity, with decreasing SD towards the extremes and symmetry around the mid-point. The SD of the total score as a function of the mean total score, shown in Supplemental Fig. 1, had a similar pattern.

**Fig. 2:**
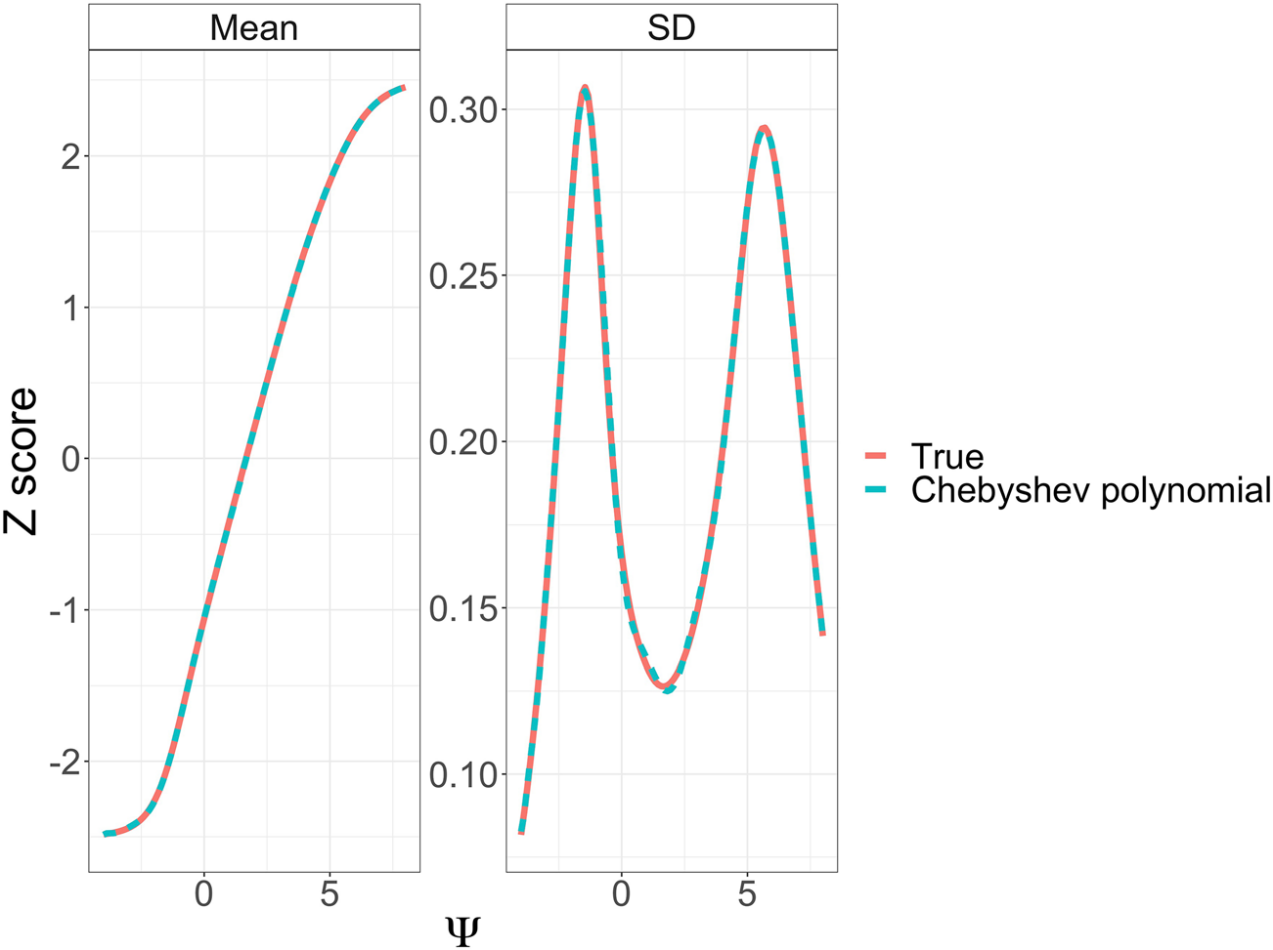
Mean Z score and SD as a function of the latent variable (Ψ) for a BI model

The Z scores increased linearly in the range of − 2 < Ψ < 5 (see Fig. 2). At the asymptotes, the relation was slightly S-shaped. The SD for the Z score also showed symmetry but with the lowest SD at the mid-point and higher SD towards the extremes. The SD of the Z score as a function of the mean Z score, shown in Supplemental Fig. 2, had a similar pattern.

#### Information Content

**Fig. 3:**
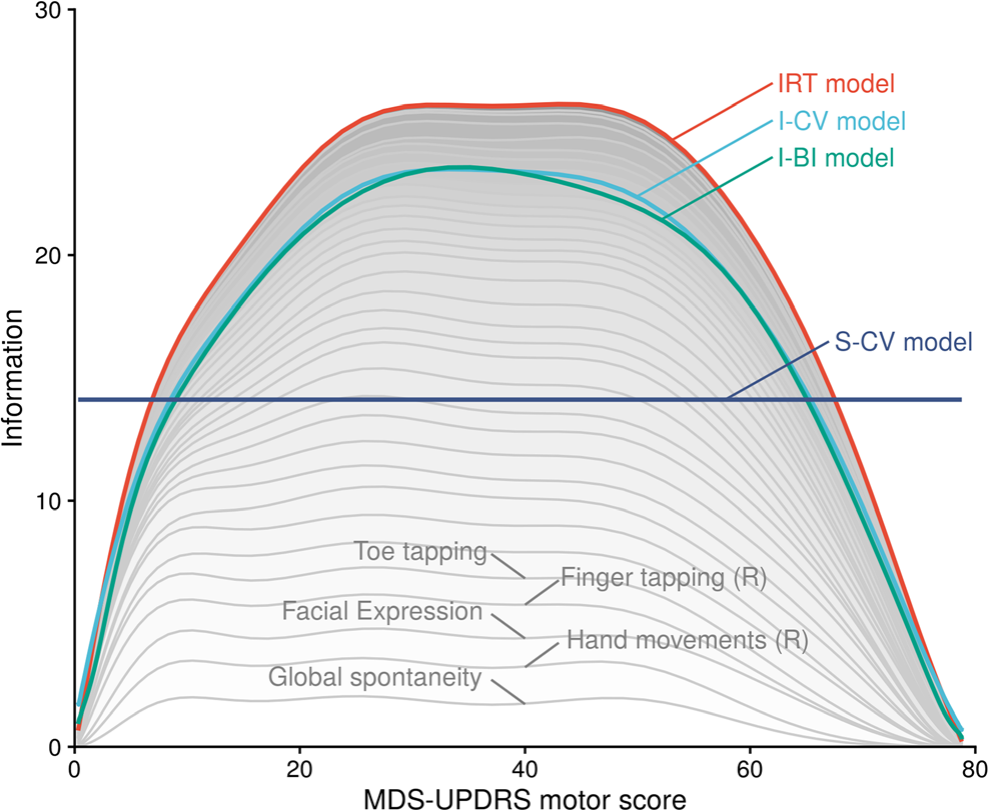
Comparison of latent variable information content *versus* total score under the IRT, the I-CV, the I-BI and the (homoscedastic) S-CV model. The grey-shaded areas illustrate that, under the IRT model, total information is the sum of information from the individual items (the 5 most informative items over the whole score range are labelled)

Figure 3 illustrates the differences in information content for the latent variable under different models. The information is calculated under the assumption that the respective model holds. The curve from the IRT model, hence, represents a theoretical upper bound for the information content as this is the true model in this case. It should be noted that the specific information value for the homoscedastic CV model is dependent on the distribution of scores in the dataset; one should therefore rather focus on the shape of the curve and not its height. The figure highlights how the homoscedastic CV model tends to underestimate the information content at the centre of the scale and overestimate it at the scale boundaries. The two IRT-informed models have similar information content curves and acknowledge the decreased information content at the scale boundaries. At the centre of the scale, where a given total score can be achieved through a larger set of response patterns than at the boundaries, the IRT model maintains the largest advantage in information content. Towards the boundaries, the difference between IRT model and IRT-informed total score models diminishes.

### Applications

#### Application to Simulated Data

In Table II, the results of TS-analysis of the simulated dataset with no disease progression are shown. For both CV and BI models, there was a significant decrease in OFV after adding *SD*(*Y*| Ψ) as a description of SD. There was no added benefit of modifying the function by multiplication or addition of extra parameters (results not shown).

**Table II:** ∆OFV for Simulated Data with No Disease Progression

Model	Standard deviation	θ	∆OFV	OFV	No. of estimated parameters	AIC
S-CV	Homoscedastic (estimated θ)	3.5	-	58,064	3	58,070
SDI-CV	Heteroscedastic (fixed *SD*(*Y*| Ψ))	-	− 299	57,765	2	57,769
S-BI	Homoscedastic (estimated θ)	0.15	-	58,118	3	58,124
SDI-BI	Heteroscedastic (fixed *SD*(*Y*| Ψ))	-	− 430	57,687	2	57,691

*AIC* Akaike information criterion, *BI* bounded integer, *CV* continuous variable, *IRT* item response theory, *OFV* objective function value, *∆OFV* difference in OFV relative to standard model, *Ψ* latent variable of IRT, *S-BI* standard BI model, *S-CV* standard CV model, *SD*(*Y*| Ψ) standard deviation from IRT model, *SDI-BI* partially (SD) IRT-informed BI model, *SDI-CV* partially (SD) IRT-informed CV model

In Table III, the results of TS-analysis of the simulated dataset with disease progression are shown. The best CV and BI models were fully IRT-informed (I-CV, I-BI) and had a 667-point and 263-point improvement in OFV compared to their respective standard models (S-CV, S-BI). The IRT-informed models also had one parameter less than the standard models, since SD was described through *SD*(*Y*| Ψ) and, thus, not estimated. The partially (SD) IRT-informed BI model (SDI-BI) had almost the same OFV as I-BI, indicating that the fit was similar on Z scale and Ψ scale, shown in Supplemental Table 1.

**Table III:** ∆OFV for Simulated Data with Disease Progression

Model	Disease progression	Standard deviation	θ	∆OFV	OFV	No. of estimated parameters	AIC
S-CV	Linear on TS	Homoscedastic (estimated θ)	3.8	-	62,171	6	62,183
I-CV	Linear on Ψ (via *E*(*TS*| Ψ))	Heteroscedastic (fixed *SD*(*Y*| Ψ))	-	− 667	61,505	5	61,515
S-BI	Linear on Z	Homoscedastic (estimated θ)	0.14	-	61,767	6	61,779
I-BI	Linear on Ψ (via *E*(*Z*| Ψ))	Heteroscedastic (fixed *SD*(*Y*| Ψ))	-	− 263	61,504	5	61,514

*AIC* Akaike information criterion, *BI* bounded integer, *CV* continuous variable, *I-BI* fully IRT-informed BI model, *I-CV* fully IRT-informed CV model, *IIV* inter-individual variability, *IRT* item response theory, *OFV* objective function value, *∆OFV* difference in OFV relative to standard model, *Ψ* latent variable of IRT, *S-BI* standard BI model, *S-CV* standard CV model, *SD*(*Y*| Ψ) standard deviation from IRT model, *TS* total score, *Z* latent variable of BI

#### Parameter Precision and Bias

Parameter precision and bias for the case of IRT model simulations and re-estimation by CV and BI models are presented in Supplemental Fig. 3. The parameters of interest were the baseline and linear slope of the IRT model (i.e. on the Ψ scale) used for simulations. The parameter precision was comparable between the models and there was no sign of bias.

#### Application to Real Data

In Table IV, the fit to the real data is shown for CV and BI models. The partially IRT-informed models are shown in Supplemental Table 2. The best CV model was fully IRT-informed (I-CV) with both a scaling factor and IIV on *SD*(*Y*| Ψ), and had a 364-point OFV improvement over the standard homoscedastic CV model (S-CV) with one additional parameter. The best BI model was the partially (SD) IRT-informed (SDI-BI) with scaling and IIV on *SD*(*Y*| Ψ), with a 309-point OFV difference compared to the standard BI model (S-BI) and one additional parameter. However, the fully IRT-informed BI model (I-BI) with the same components was comparable in fit and was chosen as the final model. The best BI model had slightly lower OFV than the best CV model.

**Table IV:** ∆OFV for Real Data

Model	Disease progression	Standard deviation	θ	IIV (%CV)	∆OFV	OFV	No. of estimated parameters	AIC
S-CV	Linear on TS	Homoscedastic (estimated θ)	5.3	-	-	18774^1^	10	18,794
I-CV	Linear on Ψ	Heteroscedastic (fixed *SD*(*Y*| Ψ))	-	-	+ 261	19,035	9	19,053
		Heteroscedastic (*SD*(*Y*| Ψ) ∙ θ)	1.4	-	− 206	18,568	10	18,588
		Heteroscedastic (*SD*(*Y*| Ψ) ∙ θ ∙ *e*^η^)	1.4	24	− 364	18411^2^	14	18,439
S-BI	Linear on Z	Homoscedastic (estimated θ)	0.22	-	-	18686^3^	10	18,706
I-BI	Linear on Ψ	Heteroscedastic (fixed *SD*(*Y*| Ψ))	-	-	+ 390	19,076	9	19,094
		Heteroscedastic (*SD*(*Y*| Ψ) ∙ θ)	1.4	-	− 97	18,590	10	18,610
		Heteroscedastic (*SD*(*Y*| Ψ) ∙ θ ∙ *e*^η^)	1.3	38	− 304	18383^4^	14	18,411

*AIC* Akaike information criterion, *BI* bounded integer, *CV* continuous variable, *%CV* coefficient of variation in percent, *I-BI* fully IRT-informed BI model, *I-CV* fully IRT-informed CV model, *IIV* inter-individual variability, *IRT* item response theory, *OFV* objective function value, *∆OFV* difference in OFV relative to standard model, *Ψ* latent variable of IRT, *S-BI* standard BI model, *S-CV* standard CV model, *SD*(*Y*| Ψ) standard deviation from IRT model, *TS* total score, *Z* latent variable of BI

^1^Base CV model

^2^Final CV model

^3^Base BI model

^4^Final BI model

**Fig. 4:**
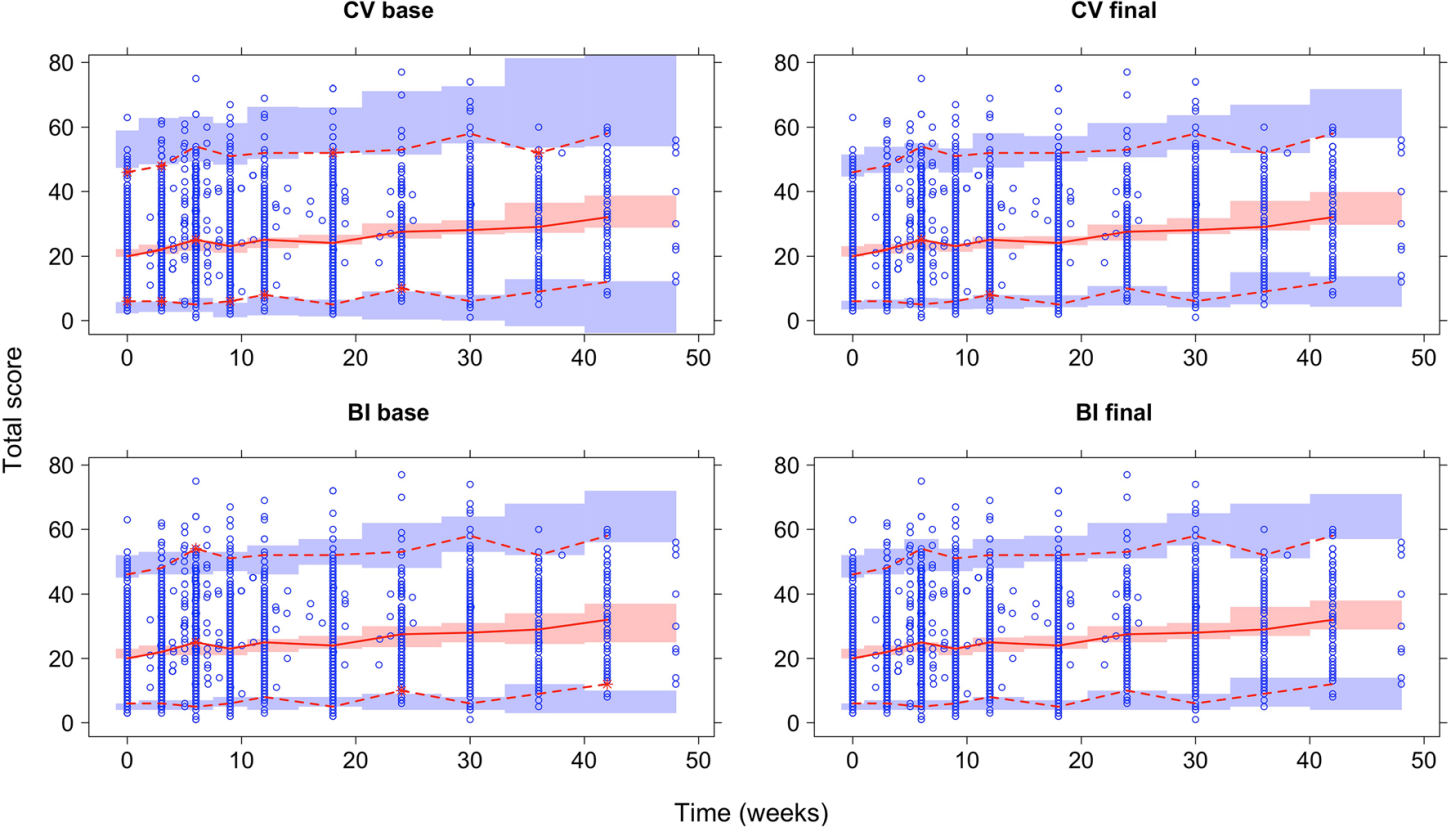
Visual predictive checks (VPC) of model fit for base and final CV and BI models for the real data. The circles represent the total score observations, the solid line represents the median, and dashed lines represent the 2.5th and 97.5th percentiles. The shaded areas represent the model predicted 95% confidence interval of the corresponding percentiles

As seen in Fig. 4, the fit for both CV and BI models was improved between the base and final model by using IRT-informed functions of mean and SD. The base CV model predicted scores outside the scale range which also resulted in wide confidence intervals for the predictions near the scale boundaries, seen in the 5th and 95th percentiles of the VPC. The final CV model has improved fit and lower uncertainty in the outer percentiles. The base BI model with homoscedastic SD respected the scale boundaries and had quite good fit, but was still improved by IRT-informed functions, with the improvements mostly seen for the median and 5th percentile of the observations/predictions. The final I-CV and I-BI had similar fit.

## DISCUSSION

The following section will discuss the general benefits of IRT-informed TS-analyses, which will be followed by the conceptual advantages shown through simulation examples, then the application to real data, and lastly some perspectives on limitations and future prospects.

The IRT-informed functions allow modellers to link IRT and TS models in a formal way. This improves the TS models, as the expected variability in scores can be better described. The functions to describe SD do not require additional parameters, yet improve the fit, as they allow the SD to vary with the disease severity and follow the nature of TS data.

The functions further make it possible to retrieve the parameters of an IRT model with a TS model, which for example could be useful for assessing potential treatment effects. The IRT-informed functions also improve the predictive performance of the models, which is especially helpful for the standard CV analyses that otherwise predict scores outside the boundaries.

The information of different model types can also be assessed via the links, and relative information can be compared. When item-level data is available in some datasets but not others, the link functions allow these data to be jointly analysed—which can help in bridging information between different studies and data bases.

### Conceptual Advantages

The mapping between Ψ and TS showed a strong nonlinear relationship, which is expected since the TS is bounded while the latent variable is not. In contrast, the Z score and Ψ mapped rather linearly in the relevant disease range, both being variables that can take on any real value. With simulated data, modelling a linear disease progression on Ψ via *E*(*Y*| Ψ) greatly improved the fit for the CV model, compared to a linear disease progression on TS. When subsequently adding *SD*(*Y*| Ψ), it further improved the fit. However, for the BI model there was only a marginal benefit of modelling via *E*(*Y*| Ψ) after adding *SD*(*Y*| Ψ).

Following the S-shaped relation between Ψ and TS, it is natural that the CV model is improved using the IRT-information for the mean when there is a linear disease progression on Ψ. This relation could also produce correlations between the baseline and slope parameters in the standard CV model since a baseline close to either boundary would mean a lower slope estimate. In the analysis of simulated data—where slope and baseline were uncorrelated—there was no large correlation estimated, likely since the baseline was in the linear range of the relation. However, correlations could be introduced or changed when transforming a variable and should be investigated.

The unexplained variability between observations was clearly not homoscedastic for either of the models. Towards the boundaries, the variability of TS was dramatically decreased almost symmetrically from the maximum value, which it reached at the midpoint. The fold difference between the maximum and minimum variability was roughly 4. For the unexplained variability of Z scores, the shape was also symmetrical, but with a nadir at the midpoint and around 3 times higher at Z ≈ − 2 or Z ≈ 2, and decreasing again at the extremes. Thus, there was a lower fold difference between the highest and lowest variability in the BI model as compared to the CV model, which might explain why the addition of *SD*(*Y*| Ψ) seemed more important for CV models than for BI models. The Z scores are not equally spaced; instead, the distance between cut-points increases as Z deviates from 0. This affects the shape of the *SD*(*Y*| Ψ) curve and explains why the function has the nadir around 0.

### Behaviour with Real Data

When applied to real data, a linear disease progression was superior on the Ψ scale over the TS scale for the best CV model (∆OFV 74), but for the best BI model, there was no difference in fit between linear disease progression on Ψ scale or Z scale (∆OFV +5). This is in line with the results from simulations, and suggests that Ψ and Z map close to each other and a linear disease progression is in better agreement with these scales than the TS scale.

For the simulated datasets, the *SD*(*Y*| Ψ) function provided the best description of SD and the fit was not improved by any additions or multiplications of the polynomial used. For the real data, however, it was better to allow individual variation of the *SD*(*Y*| Ψ) function as the description of some individuals benefitted from a reduced SD while others from an increased SD. The reduced SD for some individuals may be due to Markovian features in the responses—i.e. sequential observed scores in an individual have the same value to a higher extent than predicted by the model. Such features are well-known in categorical data analyses and have also been described, and modelled (16). Most individuals benefitted from a higher variability, since the best model had higher SD than *SD*(*Y*| Ψ) for both the CV and BI models. This indicates the presence of additional sources of variability than those accounted for in the ICCs of the IRT model. One likely explanation is that observations of different items are correlated in a way not captured by the IRT model. When simulating items of the IRT models including additional correlations across items, the overall SD was indeed different, and typically higher, than the theoretically expected (results not shown). The highest variability is seen around the mid-point of the scale and very few patients in this study were at, or close to, the scale boundaries—which explains the increased mean variability in models with homoscedastic unexplained variability. Further, describing the time trajectory with a simple model induces some model misspecification, which adds to the residual error magnitude.

Since the TS-analysis has no information about which items contributed to the score, it is natural that more observations are needed to gain the same information as an IRT model with item-level data. The item-level data is more informative because the ICCs are different for all items and different items have different information about the underlying latent variable. In contrast to the IRT model, the CV and BI models make no use of the different items ability to inform on the underlying latent variable. Indeed, the more heterogeneity in information content across items, the larger the difference between analyses on item-level and total score level (17). The standard CV model assumes constant information across all expected values. This represents an underestimation of the information in part of the scale and hence underutilizing the information, but also overestimates the information at other values, and may therefore interpret patterns that only represent noise as signals of model misspecification.

### Perspectives

We have shown the method applied to a composite scale where the TS is the sum of item scores. The Extended Disability Status Scale (EDSS) for multiple sclerosis, for example, uses a decision tree to arrive at the TS. To derive the analytical solution of the link functions and information content would in that case require a different approach. However, the option of simulating across a wide range of Ψ from the IRT model to approximate the link functions would still exist and through these simulation-based link functions arrive at the information content.

In this work we only focused on one subscale, the MDS-UPDRS motor subscale. If a scale with *N* subscales is considered, the IRT model will have *N* latent variables and *N* different TS should be characterized, which should be possible with a straightforward extension of the methodology presented here.

The degree of the polynomials used to fit the mean and SD as a function of the latent variable is a potential source of error. However, the tolerance of the Chebyshev polynomials can be adjusted to achieve a satisfactory fit, and such functionality has been built into the piraid package. When adjusting the polynomials through higher tolerance, the OFV changed only marginally in this work.

The only disease progression model that was investigated was a linear slope on the Ψ scale, as this was reported in the previously published IRT model of these data (8). Of course, in reality there may be other functions that better describe disease progression on the latent variable scale for the real data, which also had a medication effect identified as an offset effect on Ψ in the IRT model (9). In the CV model, the medication effect was also described as an offset effect, however on TS. Thus, the interpretation is different at different disease severities, due to the S-shaped relation between Ψ and TS. As the BI models mapped linearly in the relevant disease severity range (5th and 95th percentiles of Z were − 2.3 and − 0.22), the interpretation of the medication effect on Z is similar to that on Ψ. Again, more advanced functions could have been evaluated. The properties of TS models under model misspecification were not investigated here, but could be a future work. While our current approach with polynomial link functions assumes perfect knowledge of the underlying ICCs, a possible future extension could use the analytic link functions in combination with an informative prior to allow taking uncertainty into account.

There are many possible approaches to model TS data with CV models. A logit transform for the TS is one way to ensure predictions within the boundaries of the scale—however then the boundaries will only be asymptotical. In this work we only investigated untransformed TS with additive error as this is a common choice. Also for the BI model, a constant SD was used as the aim of this work was to illustrate the benefits of IRT-informed modelling of TS in a standard setting. Apart from the models mentioned above, other models for TS data (not evaluated in this work) are for example beta regression and coarsened grid models.

The usefulness of IRT-informed models to better describe the unexplained variability of composite scale endpoints is encouraging. This will facilitate analyses of TS data without the need to develop new IRT models. The impact on precision and accuracy in clinical trials is yet to be quantified, but is under investigation in a follow-up project (18). The IRT-informed functions broaden the options available to modellers dealing with TS data, and could be considered in standard analysis plans as yet another possible model to be determined from the data.

## CONCLUSIONS

IRT-informed functions provide a formal link between IRT models and TS models and allow longitudinal TS modelling to be improved without adding further parameters to the model. This approach allows information of different model types, based on item- and TS-level data, to be directly compared and their relative merits better understood. To facilitate for modellers, IRT-informed functions can be automatically generated through the piraid package.

## Supplementary Information

ESM 1 (DOCX 306 kb)

ESM 2 (DOCX 341 kb)

ESM 3 (DOCX 464 kb)

ESM 4 (DOCX 12 kb)

ESM 5 (DOCX 13 kb)

ESM 6 (PDF 48 kb)

ESM 7 (DOCX 13 kb)

ESM 8 (DOCX 14 kb)

ESM 9 (DOCX 16 kb)
