# Supplementary figures and images for: An Item Response Theory–Informed Strategy to Model Total Score Data from Composite Scales
Source: AAPS J. 2021 Mar 16;23(3):45. doi: 10.1208/s12248-021-00555-3 (PMC7966126; doi:10.1208/s12248-021-00555-3)

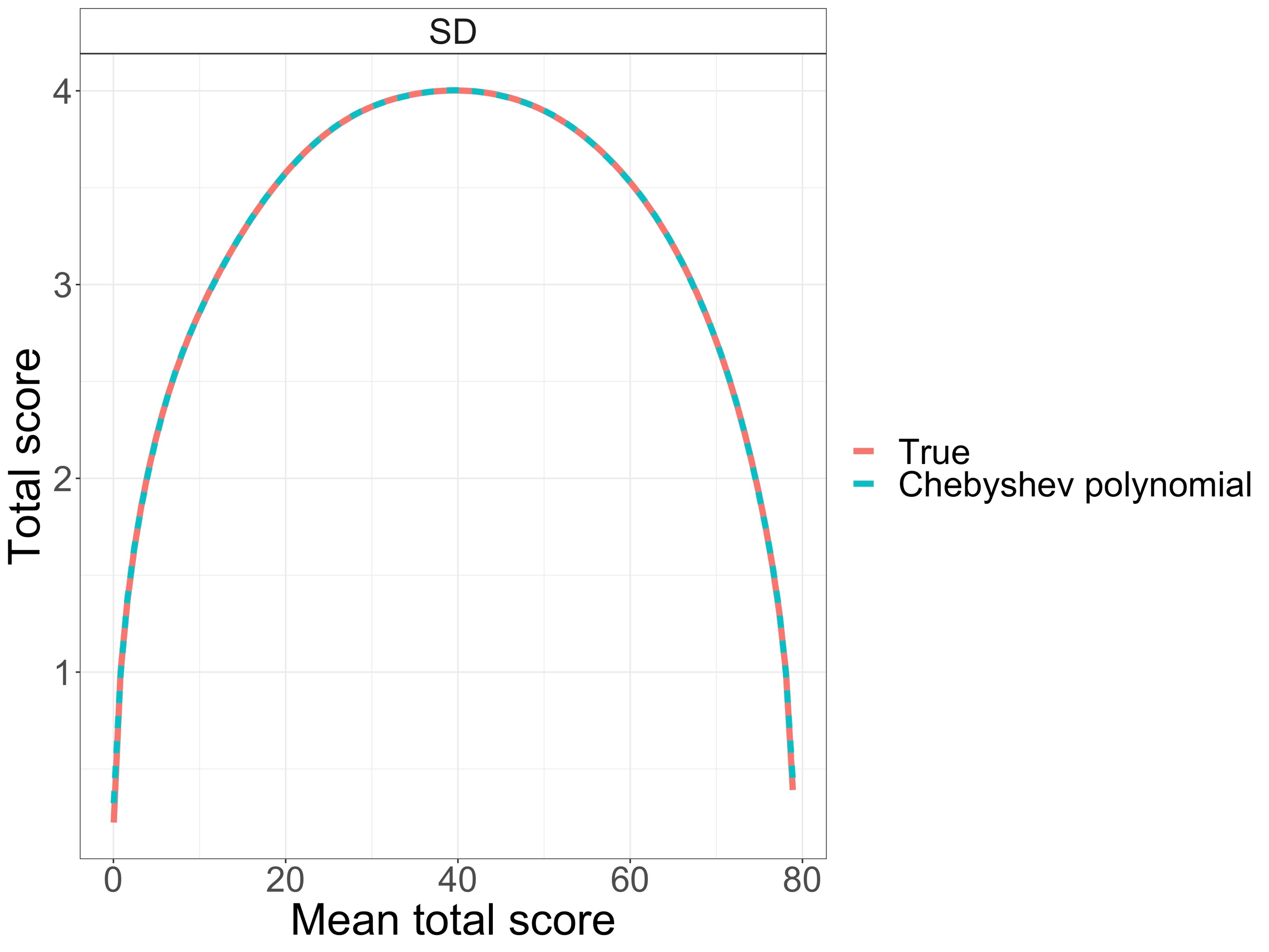


Supplemental Figure 1. CV : SD of total score as a function of mean total score.

Supplement: Supplementary file 1 — (DOCX 306 kb) [file 12248_2021_555_MOESM1_ESM.docx]

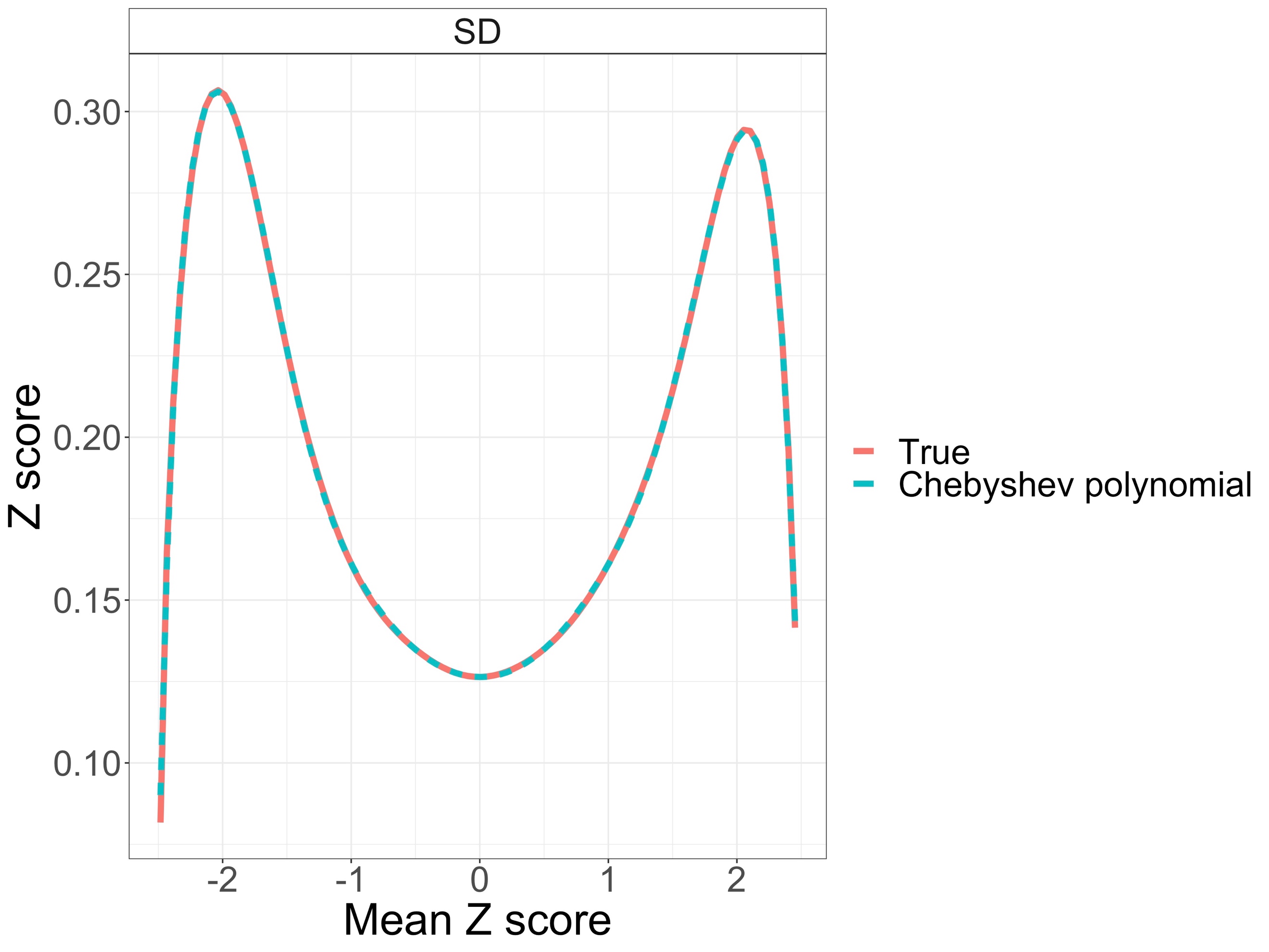
Supplemental Figure 2. BI : SD of Z score as a function of mean Z score.

Supplement: Supplementary file 2 — (DOCX 341 kb) [file 12248_2021_555_MOESM2_ESM.docx]
