# Supplementary material for: An Item Response Theory–Informed Strategy to Model Total Score Data from Composite Scales
Source: AAPS J. 2021 Mar 16;23(3):45. doi: 10.1208/s12248-021-00555-3 (PMC7966126; doi:10.1208/s12248-021-00555-3)
Supplement: Supplementary file 3 — (DOCX 464 kb) [file 12248_2021_555_MOESM3_ESM.docx]

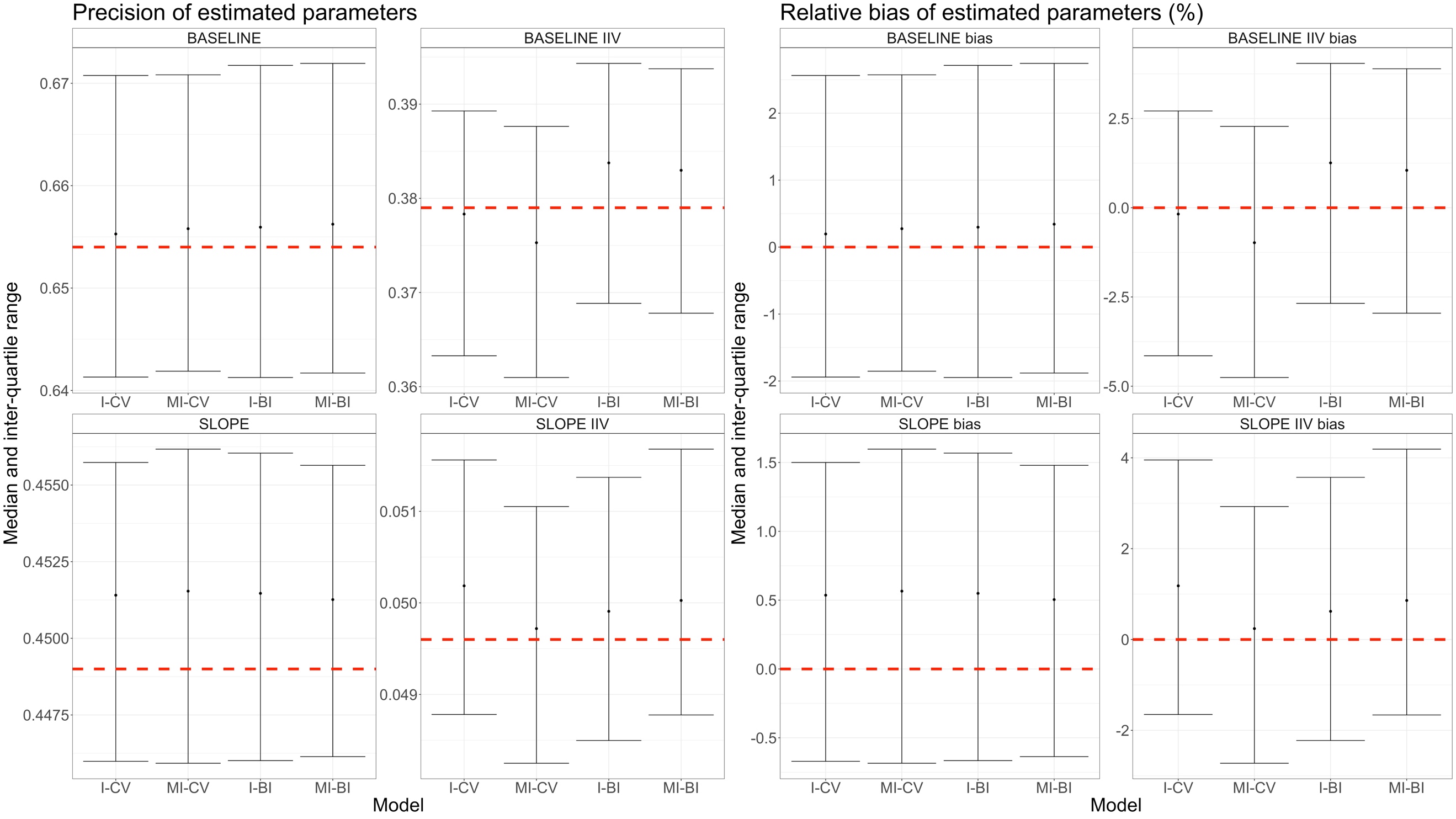


Supplemental Figure 3. Parameter precision and relative bias of CV and BI models from stochastic simulation and estimation with 100 samples. The dashed red line indicates the true parameter value.

BI, bounded integer; CV, continuous variable; I-BI, fully IRT-informed BI model; I-CV, fully IRT-informed CV model; IIV, inter-individual variability; MI-BI, partially (mean) IRT-informed BI model MI-CV, partially (mean) IRT-informed CV model.
