# Supplementary material for: An Item Response Theory–Informed Strategy to Model Total Score Data from Composite Scales
Source: AAPS J. 2021 Mar 16;23(3):45. doi: 10.1208/s12248-021-00555-3 (PMC7966126; doi:10.1208/s12248-021-00555-3)
Supplement: Supplementary file 4 — (DOCX 12 kb) [file 12248_2021_555_MOESM4_ESM.docx]

### Supplemental material 3

#### This is an R script with illustrative examples of link functions.

library(piraid)

library(ggplot2)

# Create scale

mdsupdrs <- load_scale("mds-updrs.yaml")

mds_motor <- create_subscale(scale = mdsupdrs, categories = "motor")

# create model

model <- irt_model(mds_motor) %>%

set_run_number(1) %>%

set_dataset(".../data.csv", use_path = F)

#------ CV model----

#------ CV link with LV (I-CV)

res1 <- calculate_cv_irt_link(model, psi_range = c(-4,8), lv_based = TRUE)

plot_irt_link(res1, T)

m1 <- cv_model(mds_motor, cv_irt_link = res1) %>%

set_dataset(path = "…/data.csv", use_path = F, mdv_column = "MDV")

save_model_code(m1, "run1.mod")

#------ CV link with TS (SDI-CV)

res2 <- calculate_cv_irt_link(model, psi_range=c(-4,8), lv_based = FALSE)

plot_irt_link(res2,T)

m2 <- cv_model(mds_motor, cv_irt_link = res2) %>%

set_dataset(path = "…/data.csv", use_path = F, mdv_column = "MDV")

save_model_code(m2, "run2.mod")

#------ BI model----

#------ BI link with LV (I-BI)

res3 <- calculate_bi_irt_link(model, psi_range = c(-4,8), lv_based = TRUE, approx_tol_mean = 0.01, approx_tol_sd = 0.005)

plot_irt_link(res3,T)

m3 <- bi_model(mds_motor, irt_link = res3) %>%

set_dataset(path = "…/data.csv", use_path = F, mdv_column = "MDV")

save_model_code(m3, "run3.mod")

#------ BI link with Z (SDI-BI)

res4 <- calculate_bi_irt_link(model, psi_range = c(-4,8), lv_based = FALSE)

plot_irt_link(res4,T)

m4 <- bi_model(mds_motor, irt_link = res4) %>%

set_dataset(path = "…/data.csv", use_path = F, mdv_column = "MDV")

save_model_code(m4, "run4.mod")
