# Supplementary material for: An Item Response Theory–Informed Strategy to Model Total Score Data from Composite Scales
Source: AAPS J. 2021 Mar 16;23(3):45. doi: 10.1208/s12248-021-00555-3 (PMC7966126; doi:10.1208/s12248-021-00555-3)
Supplement: Supplementary file 6 — (PDF 48 kb) [file 12248_2021_555_MOESM6_ESM.pdf]

```
$SIZES      LIM6=2892 LTH=300
$PROBLEM    IRT simulation model for MDS-UPDRS motor
$INPUT      ID TIME ITEM DV OCC
; ID   = ID number
; TIME = Time in years since start of study
; ITEM = Item of the MDS-UPDRS scale
; DV   = Patient's response to MDS-UPDRS assessment item
;        rater assessed items: 0 - 4 = category
;        (no impairment - severely impaired)
; OCC  = Occasion number

$DATA data.csv IGNORE=@
IGNORE(ITEM.LT.14)
IGNORE(ITEM.EQ.23)
IGNORE(ITEM.EQ.46)
IGNORE(ITEM.GT.49)

$ABBR DECLARE INTEGER IOCC
$ABBR DECLARE UPDRSARRM(12)

$PRED
;---------------Latent variable------------------
BSLMOTOR = THETA(1)+ETA(1)
SLPMOTOR = THETA(2)+ETA(2)
PSI = BSLMOTOR+TIME*(SLPMOTOR)

;---------------Constants to select model type---
MODEL=0
OC2=2
OC3=3
OC4=4
OC5=5

;---------------Assignment of item parameters----
IF(ITEM.EQ.14) THEN
MODEL=OC3
DIS=(1.14)
DIF1=(1.26)
DIF2=(1.18)
ENDIF

IF(ITEM.EQ.15) THEN
MODEL=OC4
DIS=(1.11)
DIF1=(1.22)
DIF2=(0.724)
```

```
DIF3=(1.11)
ENDIF

IF(ITEM.EQ.16) THEN
MODEL=OC2
DIS=(1.04)
DIF1=(2.46)
ENDIF

IF(ITEM.EQ.17) THEN
MODEL=OC3
DIS=(1.42)
DIF1=(1.16)
DIF2=(1.95)
ENDIF

IF(ITEM.EQ.18) THEN
MODEL=OC3
DIS=(2.01)
DIF1=(0.867)
DIF2=(1.53)
ENDIF

IF(ITEM.EQ.19) THEN
MODEL=OC2
DIS=(1.65)
DIF1=(1.36)
ENDIF

IF(ITEM.EQ.20) THEN
MODEL=OC4
DIS=(1.18)
DIF1=(0.263)
DIF2=(1.46)
DIF3=(1.34)
ENDIF

IF(ITEM.EQ.21) THEN
MODEL=OC3
DIS=(1.39)
DIF1=(1)
DIF2=(1.46)

ENDIF
IF(ITEM.EQ.22) THEN
MODEL=OC3
```

```
DIS=(1.24)
DIF1=(1.43)
DIF2=(2.78)
ENDIF

IF(ITEM.EQ.24) THEN
MODEL=OC3
DIS=(1.35)
DIF1=(0.82)
DIF2=(1.95)
ENDIF

IF(ITEM.EQ.25) THEN
MODEL=OC3
DIS=(1.38)
DIF1=(0.945)
DIF2=(2.32)
ENDIF

IF(ITEM.EQ.26) THEN
MODEL=OC2
DIS=(1.67)
DIF1=(2.53)
ENDIF

IF(ITEM.EQ.27) THEN
MODEL=OC3
DIS=(1.69)
DIF1=(0.758)
DIF2=(1.88)
ENDIF

IF(ITEM.EQ.28) THEN
MODEL=OC4
DIS=(2.18)
DIF1=(-0.206)
DIF2=(1.48)
DIF3=(1.52)
ENDIF

IF(ITEM.EQ.29) THEN
MODEL=OC4
DIS=(1.97)
DIF1=(0.741)
DIF2=(0.869)
DIF3=(1.64)
```

```
ENDIF

IF(ITEM.EQ.30) THEN
MODEL=OC4
DIS=(1.83)
DIF1=(-0.483)
DIF2=(1.15)
DIF3=(1.86)
ENDIF

IF(ITEM.EQ.31) THEN
MODEL=OC3
DIS=(1.66)
DIF1=(0.881)
DIF2=(1.26)
ENDIF

IF(ITEM.EQ.32) THEN
MODEL=OC4
DIS=(1.62)
DIF1=(0.54)
DIF2=(0.978)
DIF3=(1.59)
ENDIF

IF(ITEM.EQ.33) THEN
MODEL=OC3
DIS=(1.83)
DIF1=(1.42)
DIF2=(0.916)
ENDIF

IF(ITEM.EQ.34) THEN
MODEL=OC4
DIS=(2.25)
DIF1=(-0.65)
DIF2=(1.31)
DIF3=(1.1)
ENDIF

IF(ITEM.EQ.35) THEN
MODEL=OC4
DIS=(1.89)
DIF1=(0.718)
DIF2=(1.2)
DIF3=(1.28)
```

```
ENDIF

IF(ITEM.EQ.36) THEN
MODEL=OC4
DIS=(2.37)
DIF1=(-0.215)
DIF2=(1.18)
DIF3=(1.08)
ENDIF

IF(ITEM.EQ.37) THEN
MODEL=OC3
DIS=(2.05)
DIF1=(0.98)
DIF2=(1.15)
ENDIF

IF(ITEM.EQ.38) THEN
MODEL=OC4
DIS=(1.98)
DIF1=(-0.251)
DIF2=(1.22)
DIF3=(1.21)
ENDIF

IF(ITEM.EQ.39) THEN
MODEL=OC3
DIS=(2.01)
DIF1=(1.09)
DIF2=(1.19)
ENDIF

IF(ITEM.EQ.40) THEN
MODEL=OC4
DIS=(1.99)
DIF1=(-0.334)
DIF2=(1.35)
DIF3= (1.24)
ENDIF

IF(ITEM.EQ.41) THEN
MODEL=OC4
DIS=(1.45)
DIF1=(0.89)
DIF2=(1.53)
DIF3=(1.67)
```

```
ENDIF

IF(ITEM.EQ.42) THEN
MODEL=OC4
DIS=(1.96)
DIF1=(0.364)
DIF2=(1.31)
DIF3= (1.4)
ENDIF

IF(ITEM.EQ.43) THEN
MODEL=OC3
DIS=(1.79)
DIF1=(1.37)
DIF2=(1.32)
ENDIF

IF(ITEM.EQ.44) THEN
MODEL=OC3
DIS=(1.36)
DIF1=(2.02)
DIF2=(1.68)
ENDIF

IF(ITEM.EQ.45) THEN
MODEL=OC3
DIS=(1.42)
DIF1=(0.437)
DIF2=(2.44)
ENDIF

IF(ITEM.EQ.47) THEN
MODEL=OC3
DIS=(0.902)
DIF1=(3.28)
DIF2=(1.38)
ENDIF

IF(ITEM.EQ.48) THEN
MODEL=OC3
DIS=(1.29)
DIF1=(0.447)
DIF2=(2.05)
ENDIF

IF(ITEM.EQ.49) THEN
```

```
MODEL=OC4
DIS=(2.76)
DIF1=(-0.172)
DIF2=(1.1)
DIF3=(1.22)
ENDIF

;--------Ordered categorical data model----------
IF(MODEL.EQ.OC2) THEN
DIFG1=DIF1

PGE1=EXP(DIS*(PSI-DIFG1))/(1+EXP(DIS*(PSI-DIFG1)))

P0=1-PGE1
P1=PGE1

ENDIF

IF(MODEL.EQ.OC2.AND.DV.EQ.0) P=P0
IF(MODEL.EQ.OC2.AND.DV.GE.1) P=P1


IF(P.LT.1E-16) P=1E-16
IF(P.GT.(1-1E-16)) P=1-1E-16
IF(MODEL.EQ.OC2) Y=-2*LOG(P)

;--------Ordered categorical data model----------
IF(MODEL.EQ.OC3) THEN
DIFG1=DIF1
DIFG2=DIFG1+DIF2

PGE1=EXP(DIS*(PSI-DIFG1))/(1+EXP(DIS*(PSI-DIFG1)))
PGE2=EXP(DIS*(PSI-DIFG2))/(1+EXP(DIS*(PSI-DIFG2)))

P0=1-PGE1
P1=PGE1-PGE2
P2=PGE2
ENDIF

IF(MODEL.EQ.OC3.AND.DV.EQ.0) P=P0
IF(MODEL.EQ.OC3.AND.DV.EQ.1) P=P1
IF(MODEL.EQ.OC3.AND.DV.GE.2) P=P2


IF(P.LT.1E-16) P=1E-16
IF(P.GT.(1-1E-16)) P=1-1E-16
```

```
IF(MODEL.EQ.OC3) Y=-2*LOG(P)

;--------Ordered categorical data model----------
IF(MODEL.EQ.OC4) THEN
DIFG1=DIF1
DIFG2=DIFG1+DIF2
DIFG3=DIFG2+DIF3

PGE1=EXP(DIS*(PSI-DIFG1))/(1+EXP(DIS*(PSI-DIFG1)))
PGE2=EXP(DIS*(PSI-DIFG2))/(1+EXP(DIS*(PSI-DIFG2)))
PGE3=EXP(DIS*(PSI-DIFG3))/(1+EXP(DIS*(PSI-DIFG3)))

P0=1-PGE1
P1=PGE1-PGE2
P2=PGE2-PGE3
P3=PGE3
ENDIF

IF(MODEL.EQ.OC4.AND.DV.EQ.0) P=P0
IF(MODEL.EQ.OC4.AND.DV.EQ.1) P=P1
IF(MODEL.EQ.OC4.AND.DV.EQ.2) P=P2
IF(MODEL.EQ.OC4.AND.DV.GE.3) P=P3

IF(P.LT.1E-16) P=1E-16
IF(P.GT.(1-1E-16)) P=1-1E-16
IF(MODEL.EQ.OC4) Y=-2*LOG(P)

;--------Ordered categorical data model----------
IF(MODEL.EQ.OC5) THEN
DIFG1=DIF1
DIFG2=DIFG1+DIF2
DIFG3=DIFG2+DIF3
DIFG4=DIFG3+DIF4

PGE1=EXP(DIS*(PSI-DIFG1))/(1+EXP(DIS*(PSI-DIFG1)))
PGE2=EXP(DIS*(PSI-DIFG2))/(1+EXP(DIS*(PSI-DIFG2)))
PGE3=EXP(DIS*(PSI-DIFG3))/(1+EXP(DIS*(PSI-DIFG3)))
PGE4=EXP(DIS*(PSI-DIFG4))/(1+EXP(DIS*(PSI-DIFG4)))

P0=1-PGE1
P1=PGE1-PGE2
P2=PGE2-PGE3
P3=PGE3-PGE4
P4=PGE4
ENDIF
```

```
IF(MODEL.EQ.OC5.AND.DV.EQ.0) P=P0
IF(MODEL.EQ.OC5.AND.DV.EQ.1) P=P1
IF(MODEL.EQ.OC5.AND.DV.EQ.2) P=P2
IF(MODEL.EQ.OC5.AND.DV.EQ.3) P=P3
IF(MODEL.EQ.OC5.AND.DV.EQ.4) P=P4

IF(P.LT.1E-16) P=1E-16
IF(P.GT.(1-1E-16)) P=1-1E-16
IF(MODEL.EQ.OC5) Y=-2*LOG(P)

;--------Simulation code-------------------------
IF(ICALL.EQ.4) THEN
ISIM=IREP

IF(MODEL.EQ.OC2) THEN
CALL RANDOM (2,R)
SDV=0
IF(R.LT.PGE1) SDV=1
ENDIF

IF(MODEL.EQ.OC3) THEN
CALL RANDOM (2,R)
SDV=0
IF(R.LT.PGE1) SDV=1
IF(R.LT.PGE2) SDV=2
ENDIF

IF(MODEL.EQ.OC4) THEN
CALL RANDOM (2,R)
SDV=0
IF(R.LT.PGE1) SDV=1
IF(R.LT.PGE2) SDV=2
IF(R.LT.PGE3) SDV=3
ENDIF

IF(MODEL.EQ.OC5) THEN
CALL RANDOM (2,R)
SDV=0
IF(R.LT.PGE1) SDV=1
IF(R.LT.PGE2) SDV=2
IF(R.LT.PGE3) SDV=3
IF(R.LT.PGE4) SDV=4
ENDIF
DV=SDV

ENDIF
```

```
;--------Calculation of MDS-UPDRS motor score----
IF(NEWIND.NE.2) THEN
UPDRSARRM(1)=0
UPDRSARRM(2)=0
UPDRSARRM(3)=0
UPDRSARRM(4)=0
UPDRSARRM(5)=0
UPDRSARRM(6)=0
UPDRSARRM(7)=0
UPDRSARRM(8)=0
UPDRSARRM(9)=0
UPDRSARRM(10)=0
UPDRSARRM(11)=0
UPDRSARRM(12)=0
ENDIF

IOCC=0
"IOCC=INT(OCC)

IF(ITEM.GT.13.AND.ITEM.NE.46.AND.ITEM.NE.23.AND.ITEM.LT.50) THEN
UPDRSARRM(IOCC)=UPDRSARRM(IOCC) + DV
ENDIF

UPDRSMOTOR=UPDRSARRM(IOCC)

;--------Baseline and slope----------------------
$THETA
... ; 1 BSLMOTOR
... ; 2 SLPMOTOR

;--------Random effects for baseline and slope---
$OMEGA
... ; 1 BSLMOTOR_IIV
... ; 2 SLPMOTOR_IIV

$SIMULATION (...) (... UNIFORM) NOPREDICTION
            ONLYSIMULATION SUBPROBLEMS=1
$TABLE  ID TIME ITEM DV UPDRSMOTOR
        NOAPPEND NOHEADER NOPRINT FILE=... FORMAT=,1PE11.4
```
