# Supplementary material for: An Item Response Theory–Informed Strategy to Model Total Score Data from Composite Scales
Source: AAPS J. 2021 Mar 16;23(3):45. doi: 10.1208/s12248-021-00555-3 (PMC7966126; doi:10.1208/s12248-021-00555-3)
Supplement: Supplementary file 7 — (DOCX 13 kb) [file 12248_2021_555_MOESM7_ESM.docx]

### Supplemental material 6

#### Information for IRT model

The Fisher information for the IRT model was calculated according to:

$$\mathcal{I}_{IRT}\left( \Psi\right)=\sum_{m=1}^{M} \sum_{s=0}^{S_{m}} P\left( Y_{m}=s | \Psi\right)\frac{d^{2}P\left( Y_{m}=s | \Psi\right)}{d\Psi^{2}}$$

where $P\left( Y_{m}=s | \Psi\right)$ was defined as:

$$P\left( Y_{m}=0 | \Psi\right)=1-P\left( Y_{m}\geq1 | \Psi\right)$$

$$P\left( Y_{m}=s | \Psi\right)= P\left( Y_{m}\geq s | \Psi\right)-P\left( Y_{m}\geq s+1 | \Psi\right)$$

and $P\left( Y_{m}\geq s | \Psi\right)$ was calculated from the item characteristic functions of the model.

#### Information for the fully IRT-informed CV model (I-CV)

Let $p_{\mu}\left( \Psi\right)$ and $p_{\sigma}\left( \Psi\right)$ denote the polynomials approximating the mean and SD of the observations from the IRT model. The Fisher information for the fully IRT-informed CV model (I-CV) was calculated as:

$$\mathcal{I}_{CV}\left( \Psi\right)=\frac{p_{\mu}^{'}\left( \Psi\right)^{2}}{p_{\sigma}\left( \Psi\right)^{2}}+2\frac{\left( p_{\sigma}\left( \Psi\right)p_{\sigma}^{'}\left( \Psi\right) \right)^{2}}{p_{\sigma}\left( \Psi\right)^{4}}$$

#### Information for the fully IRT-informed BI model (I-BI)

Let $p_{\bar{\mu}}\left( \Psi\right)$ and $p_{\bar{\sigma}}\left( \Psi\right)$ denote the polynomials approximating the expected value and SD of the Z score according to the IRT model. The Fisher information for the fully IRT-informed BI (I-BI) model was calculated as:

$$\mathcal{I}_{BI}\left( \Psi\right)=E\left( {\frac{d\log\mathcal{L}\left( \Psi\right)}{d\Psi}}^{2} \right)$$

$$\frac{d\log\mathcal{L}\left( \Psi\right)}{d\Psi}=\frac{1}{P\left( Y=s | \Psi\right)}\left( \phi\left( z_{l},p_{\bar{\mu}}\left( \Psi\right),p_{\bar{\sigma}}\left( \Psi\right) \right)-\phi\left( z_{l+1},p_{\bar{\mu}}\left( \Psi\right),p_{\bar{\sigma}}\left( \Psi\right) \right) \right)\frac{(p_{\bar{\mu}}\left( \Psi\right)-\Psi){p'}_{\bar{\sigma}}\left( \Psi\right)-p_{\bar{\sigma}}\left( \Psi\right){p'}_{\bar{\mu}}\left( \Psi\right)}{p_{\bar{\sigma}}\left( \Psi\right)^{2}}$$

where $\phi(x,\mu,\sigma)$ denotes the probability density function of the normal distribution with mean $\mu$ and SD $\sigma$.
