# Supplementary material for: An Item Response Theory–Informed Strategy to Model Total Score Data from Composite Scales
Source: AAPS J. 2021 Mar 16;23(3):45. doi: 10.1208/s12248-021-00555-3 (PMC7966126; doi:10.1208/s12248-021-00555-3)
Supplement: Supplementary file 8 — (DOCX 14 kb) [file 12248_2021_555_MOESM8_ESM.docx]

Supplemental Table 1. ∆OFV for simulated data with disease progression

| Model | Disease progression | Standard deviation | θ | ∆OFV | OFV | No. of estimated parameters | AIC |
| --- | --- | --- | --- | --- | --- | --- | --- |
| S-CV | Linear on TS | Homoscedastic (estimated θ) | 3.8 | - | 62171 | 6 | 62183 |
| SDI-CV |  | Heteroscedastic (fixed $SD(Y\vert\Psi)$) | - | -13 | 62158 | 5 | 62168 |
| MI-CV | Linear on $\Psi$ (via $\left( TS \vert\Psi\right)$) | Homoscedastic (estimated θ) | 3.7 | -469 | 61701 | 6 | 61713 |
| I-CV |  | Heteroscedastic (fixed $SD(Y\vert\Psi)$) | - | -667 | 61505 | 5 | 61515 |
| S-BI | Linear on Z | Homoscedastic (estimated θ) | 0.14 | - | 61767 | 6 | 61779 |
| SDI-BI |  | Heteroscedastic (fixed $SD(Y\vert\Psi)$) | - | -256 | 61511 | 5 | 61521 |
| MI-BI | Linear on $\Psi$ (via $E\left( Z \vert\Psi\right)$) | Homoscedastic (estimated θ) | 0.14 | -12 | 61756 | 6 | 61768 |
| I-BI |  | Heteroscedastic (fixed $SD(Y\vert\Psi)$) | - | -263 | 61504 | 5 | 61514 |

AIC, Akaike information criterion; BI, bounded integer; CV, continuous variable; I-BI, fully IRT-informed BI model; I-CV, fully IRT-informed CV model, IIV, inter-individual variability; IRT, item response theory; MI-BI, partially (mean) IRT-informed BI model; MI-CV, partially (mean) IRT-informed CV model; OFV, objective function value; ∆OFV, difference in OFV relative to standard model; $\Psi$, latent variable of IRT; S-BI, standard BI model; S-CV, standard CV model;$SD(Y|\Psi)$, standard deviation from IRT model; SDI-BI, partially (SD) IRT-informed BI model; SDI-CV, partially (SD) IRT-informed CV model; TS, total score; Z, latent variable of BI.
