# Supplementary material for: An Item Response Theory–Informed Strategy to Model Total Score Data from Composite Scales
Source: AAPS J. 2021 Mar 16;23(3):45. doi: 10.1208/s12248-021-00555-3 (PMC7966126; doi:10.1208/s12248-021-00555-3)
Supplement: Supplementary file 9 — (DOCX 16 kb) [file 12248_2021_555_MOESM9_ESM.docx]

Supplemental Table 2. ∆OFV for real data

| Model | Disease progression | Standard deviation | θ | IIV (%CV) | ∆OFV | OFV | No. of estimated parameters | AIC |
| --- | --- | --- | --- | --- | --- | --- | --- | --- |
| S-CV | Linear on TS | Homoscedastic (estimated θ) | 5.3 | - | - | 18774^1^ | 10 | 18794 |
| SDI-CV |  | Heteroscedastic (fixed $SD(Y\vert\Psi)$) | - | - | +372 | 19146 | 9 | 19164 |
|  |  | Heteroscedastic ($SD(Y\vert\Psi)\cdot\theta$) | 1.4 | - | -134 | 18640 | 10 | 18660 |
|  |  | Heteroscedastic ($SD(Y\vert\Psi)\cdot\theta\cdot e$^η^) | 1.4 | 25 | -289 | 18485 | 14 | 18513 |
| MI-CV | Linear on $\Psi$ | Homoscedastic (estimated θ) | 5.2 | 41 | -61 | 18713 | 10 | 18733 |
| I-CV |  | Heteroscedastic (fixed $SD(Y\vert\Psi)$) | - | - | +261 | 19035 | 9 | 19053 |
|  |  | Heteroscedastic ($SD(Y\vert\Psi)\cdot\theta$) | 1.4 | - | -206 | 18568 | 10 | 18588 |
|  |  | Heteroscedastic ($SD(Y\vert\Psi)\cdot\theta\cdot e$^η^) | 1.4 | 24 | -364 | 18411^2^ | 14 | 18439 |
| S-BI | Linear on Z | Homoscedastic (estimated θ) | 0.22 | - | - | 18686^3^ | 10 | 18706 |
| SDI-BI |  | Heteroscedastic (fixed $SD(Y\vert\Psi)$) | - | - | +385 | 19071 | 9 | 19089 |
|  |  | Heteroscedastic ($SD(Y\vert\Psi)\cdot\theta$) | 1.4 | - | -102 | 18584 | 10 | 18604 |
|  |  | Heteroscedastic ($SD(Y\vert\Psi)\cdot\theta\cdot e$^η^) | 1.3 | 38 | -309 | 18378 | 14 | 18406 |
| MI-BI | Linear on $\Psi$ | Homoscedastic (estimated θ) | 0.20 | - | -133 | 18553 | 10 | 18573 |
| I-BI |  | Heteroscedastic (fixed $SD\left( Y \vert\Psi\right)$) | - | - | +390 | 19076 | 9 | 19094 |
|  |  | Heteroscedastic ($SD(Y\vert\Psi)\cdot\theta$) | 1.4 | - | -97 | 18590 | 10 | 18610 |
|  |  | Heteroscedastic ($SD(Y\vert\Psi)\cdot\theta\cdot e$^η^) | 1.3 | 38 | -304 | 18383^4^ | 14 | 18411 |

AIC, Akaike information criterion; BI, bounded integer; CV, continuous variable; %CV, Coefficient of variation in percent; I-BI, fully IRT-informed BI model; I-CV, fully IRT-informed CV model, IIV, inter-individual variability; IRT, item response theory; MI-BI, partially (mean) IRT-informed BI model; MI-CV, partially (mean) IRT-informed CV model; OFV, objective function value; ∆OFV, difference in OFV relative to standard model; $\Psi$, latent variable of IRT; S-BI, standard BI model; S-CV, standard CV model;$SD(Y|\Psi)$, standard deviation from IRT model; SDI-BI, partially (SD) IRT-informed BI model; SDI-CV, partially (SD) IRT-informed CV model; TS, total score; Z, latent variable of BI.

^1^Base CV model

^2^Final CV model

^3^Base BI model

^4^Final BI model
